# Anticipatory postural control in adaptation of goal-directed lower extremity movements

**DOI:** 10.1038/s41598-024-54672-y

**Published:** 2024-02-20

**Authors:** Mai Moriyama, Motoki Kouzaki, Shota Hagio

**Affiliations:** 1 Laboratory of Neurophysiology, Graduate School of Human and Environmental Studies, Kyoto University, Kyoto, Japan; 2 Research Fellow of the Japan Society for the Promotion of Science, Tokyo, Japan; 3 Unit of Synergetic Studies for Space, Kyoto University, Kyoto, Japan; 4 Laboratory of Motor Control and Learning, Graduate School of Human and Environmental Studies, Kyoto University, Kyoto, Japan

**Keywords:** Motor control, Learning and memory

## Abstract

Skilled football players can adapt their kicking movements depending on external environments. Predictive postural control movements, known as anticipatory postural adjustments (APAs), are needed preceding kicking movements to precisely control them while maintaining a standing posture only with the support leg. We aimed to clarify APAs of the support leg in the process of adaptation of goal-directed movements with the lower limb. Participants replicated ball-kicking movements such that they reached a cursor, representing a kicking-foot position towards a forward target while standing with the support leg. APAs were observed as the centre of pressure of the support leg shifted approximately 300 ms in advance of the onset of movement of the kicking foot. When the cursor trajectory of the kicking foot was visually rotated during the task, the kicking-foot movement was gradually modified to reach the target, indicating adaptation to the novel visuomotor environment. Interestingly, APAs in the mediolateral direction were also altered following the change in kicking-foot movements. Additionally, the APAs modified more slowly than the kicking-foot movements. These results suggest that flexible changes in predictive postural control might support the adaptation of goal-directed movements of the lower limb.

## Introduction

Skilled football players can kick a ball into the goal precisely not only in a familiar condition but also in unfamiliar grounds or windy conditions. How do they adapt their movements to ever-changing environments? Ball-kicking movement, one of the representative movements with the lower limbs, requires goal-directed control of the kicking leg. At the same time, the other leg contributes to postural control, which is the primary role of the lower limbs in stabilizing or translating the whole body centre of mass (COM) during daily living activities^1,2^. Kicking movements considerably demand balance control^3,4^, and it has been reported that single-leg balance correlates with kicking accuracy^5^. Hence, precise ball-kicking movement demands coordination of the goal-directed control of the kicking leg and postural control from the support leg.

In order to accomplish desired movements, motor adaptation is an essential ability for human. This is because both the environment and the human body are constantly changing, so that an invariable motor command would result in different movements. Furthermore, because real-world movements such as football are dynamic and involve the use of the whole body^6^, the process of motor adaptation is extremely complex. Therefore, motor adaptation has been investigated using laboratory-based experimental tasks in highly controlled conditions. The study of the sensorimotor adaptation of goal-directed movements has commonly employed arm-reaching tasks in which the cursor trajectory is rotated relative to the hand or the dynamics of the field are modified through a handle held by the participants^7,8^. These studies revealed that goal-directed movements with the upper limb can be adapted to a novel visuomotor environment or novel dynamics. Recently, we demonstrated that goal-directed movements with the lower limb can also be adapted to a novel visuomotor environment in which the trajectory of the kicked ball is rotated from the actual trajectory^9^. However, how posture is controlled in the support leg during the adaptation of the kicking leg remains unclear.

When we conduct goal-directed movements while standing, postural muscles are activated in advance of the voluntary movement; consequently, the centre of pressure (COP) changes prior to movement onset^10–13^. Such predictive controls are known as anticipatory postural adjustments (APAs). APAs are driven by feedforward mechanisms and are presumed to operate to maintain postural equilibrium by controlling the COM against the shift in dynamics caused by the movement^14,15^. Alternatively, it has also been shown that while reaching movements were accompanied by large trunk motion, postural muscles were activated to assist the desired voluntary action rather than stabilizing the COM^16,17^. While APAs precede familiar and predictable movements, a study examining arm-reaching with postural control showed that anticipatory postural control can be learned with hand movements when participants were exposed to novel dynamics through holding a handle on a robot arm^18–20^. Given that ball-kicking movements are conducted with an unstable single-leg stance, even small APAs could influence kicking movements more than arm reaching movements in a stable standing posture. Therefore, it is possible that anticipatory postural control in the support leg would play a key role in the process of the adaptation of the kicking-leg movements to a novel visuomotor environment. Moreover, previous studies on APAs during gait initiation^1^ and ball-kicking movements^21^ demonstrated that the direction of the COP displacement just before the lower-limb swing is opposite in the anteroposterior direction, although ball-kicking movements are the same with walking in that both involve swinging the lower limb. Thus, postural control in ball-kicking movements would be different from that of arm reaching movements or gait action.

The purpose of this study was to investigate how a standing posture is controlled during the adaptation of goal-directed movements in the lower limb, focusing on anticipatory postural control. To this end, COP displacement in the support leg was measured when the visual feedback of the endpoint of the kicking leg was rotated relative to the actual trajectory^9^. This study conducted exploratory examination of the anticipatory postural control in the support leg and quantified its behaviour during visuomotor adaptation in the kicking leg movements. Subsequently, we examined APAs defined as COP displacement before the initiation of the kicking movement during visuomotor adaptation in the kicking leg movements.

## Results

**Figure 1:**
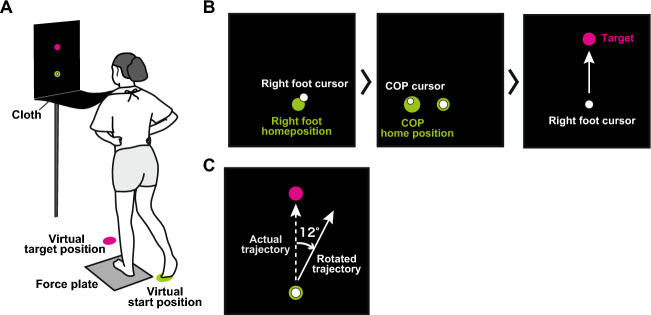
Experimental setup. (**A**) Participants swung their right foot while standing barefoot with their left foot on a force plate and with their hands on their hips.﻿ A cloth prevented them from seeing their foot. They were asked to swing their right foot and shoot a target shown in the display. (**B**) The display shown in front of the participants. At the beginning of each trial, a white cursor representing the right foot position on the horizontal plane and a green circle of its home position were displayed on the screen. Once the right foot cursor was placed within the home position, another white cursor and green circle were displayed, corresponding to the COP position of the support leg and its home position, respectively. After both cursors were held in the home positions, a target appeared, and participants were asked to start the kicking movement. (**C**) The rotated trajectory of the cursor during the perturbation period. The trajectory of the right foot was rotated to 12° clockwise from the actual trajectory.

Participants performed a shooting task with their right foot while standing with their left foot on a force plate (Fig. 1A). The right foot position on the horizontal plane was displayed as a cursor on the screen (Fig. 1B). After the right foot and the COP of the support leg were held in the home positions, a target appeared, and participants reached the right-foot cursor to the target by kicking movements. The experiment consisted of three phases: baseline, perturbation, and washout periods. During the perturbation period, the cursor trajectory of the right foot was rotated clockwise relative to the actual trajectory by 12° (Fig. 1C).

### Typical behaviour of the kicking-leg movements and the COP of the support leg

**Figure 2:**
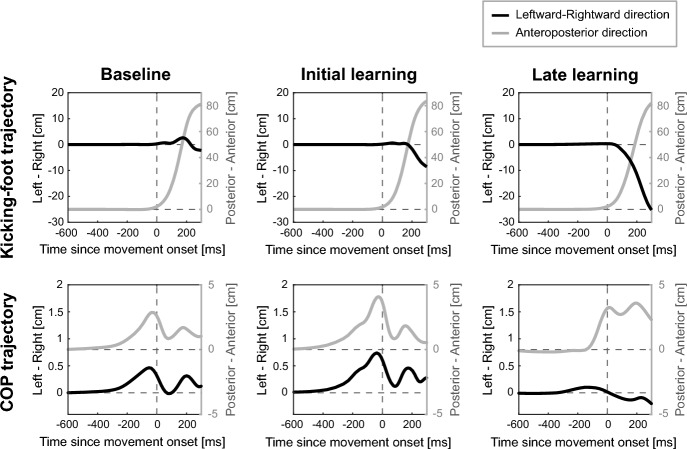
Trajectories of the kicking foot and the COP. Trajectories of the kicking foot (the upper row) and the COP of the support leg (the lower row) for a representative participant are shown. Each trajectory is shown from 600 ms before the movement onset of the kicking foot until 300 ms after the movement onset. These trajectories are the mean trajectories of the last 50 baseline trials (baseline), the initial 10 perturbation trials (initial learning) and the last 50 perturbation trials (late learning).

**Figure 3:**
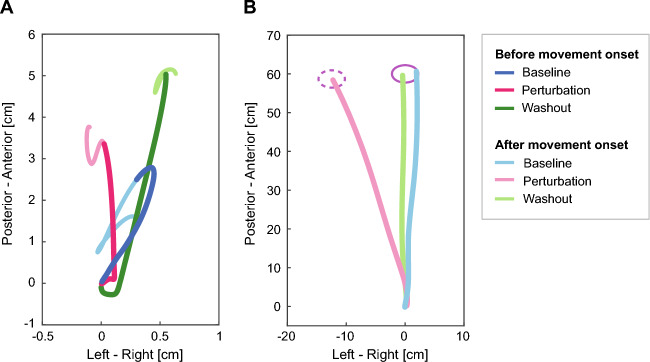
Trajectories of the COP and the kicking foot in the late trials of each phase. Both figures show mean trajectories of the COP and the kicking foot in the last 50 trials of the baseline (blue lines), perturbation (pink lines) and washout period (green lines) for the same representative participant as Fig. 2. (**A**) Trajectories of the COP of the support leg from 400 ms before movement onset until movement onset of the kicking foot (deep-coloured lines) and from movement onset until 200 ms after movement onset (pale-coloured lines), which is approximately the moment when the cursor reached the target. (**B**) Trajectories of the kicking foot on the horizontal plane from the home position until the right foot reached the target distance. A pink circle with a solid line indicates the virtual target location, and a pink circle with a dotted line shows where to kick to reach the cursor to the target in the perturbation period, during which the cursor trajectory was rotated by 12° clockwise.

Figures 2 and 3 show the trajectories of the kicking foot and the COP of the support leg from a representative participant. During the baseline period, the kicking foot moved to the forwards target and shot the target in approximately 200 ms (upper left in Fig. 2). Regarding the posture control of the support leg, the COP of this leg began deviating from the original position towards the kicking leg in the mediolateral direction and forwards in the anteroposterior direction (lower left in Fig. 2) in advance of the kicking foot movement onset by approximately 300 ms, demonstrating an anticipatory postural response in the COP of the support leg when performing ball-kicking movements.

During the early phase of the perturbation period, kicking-leg movements gradually changed leftward to cancel the clockwise rotation of the cursor trajectory (the upper row in Fig. 2). Along with the leftward change in the kicking foot movements, the rightward displacement of the COP before the foot movement onset was gradually reduced (the lower row in Fig. 2). At the end of the perturbation period, participants kicked leftward relative to the actual target position (Fig. 3B). In addition, the direction of the COP trajectories before the movement onset inclined to the left compared with the baseline COP trajectory (Fig. 3A).

When the perturbation was suddenly removed in the washout period, the foot trajectories returned to the baseline trajectory by the end of this period (Fig. 3B). Moreover, rightward displacement of the COP was observed after the removal of the perturbation (Fig. 3A), indicating that the mediolateral displacement of the COP returned to baseline, as did the goal-directed movement of the kicking leg.

### Time course of learning of the kicking movements and APAs

**Figure 4:**
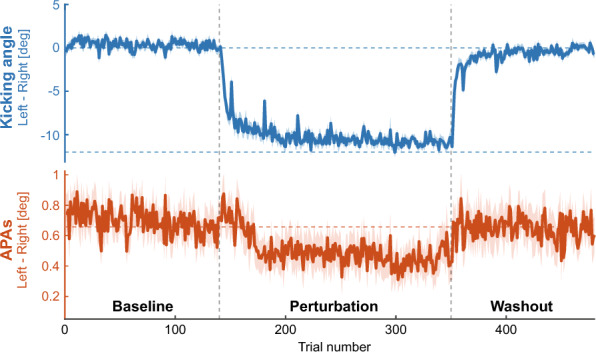
Time course of the kicking angle and the APAs. The blue and orange lines show the kicking angle and the APAs averaged across participants, respectively. A positive value of both corresponds to the rightward deviation and vice versa. The shaded area represents the standard error of the mean. The experiment consisted of three phases involving 480 trials in total: a baseline period (140 trials), a perturbation period (210 trials), and a washout period (130 trials). A horizontal blue dotted line at the top of the figure indicates the angle of 0°, and another horizontal blue dotted line in the middle of the figure indicates the angle of − 12°, which is the angle that perfectly compensates for the perturbation. A horizontal orange dotted line indicates the mean APAs of the last 50 baseline trials.

The kicking angle was calculated ﻿as the angular difference between the directions of the target and the right-foot position when the foot-movement distance exceeded the target distance (60 cm) to measure the goal-directed movements of the kicking leg for each trial. The APAs were measured as a mediolateral COP displacement at 50 ms before the kicking foot started to move because changes in the kicking-foot movements induced by the visuomotor rotation during the perturbation period were mainly in the mediolateral direction. Figure 4 shows the mean kicking angle and the mean APAs for all participants in all trials. One-way repeated-measures ANOVAs revealed that both the kicking angle and the APAs changed depending on the phase of the experiment: baseline, initial learning, late learning and initial washout trials (F(3, 54) = 169.5, *p* < 0.001, partial ${\eta }^{2}$ = 0.90; F(3, 54) = 6.97, *p* < 0.001, partial ${\eta }^{2}$ = 0.28, respectively).

**Figure 5:**
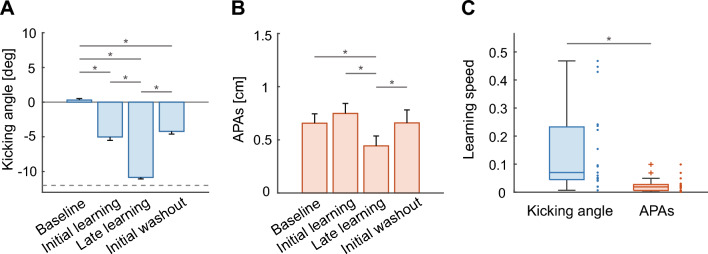
Kicking angle and APAs for each participant. (**A**,** B**) Comparison of the kicking angle and the APAs for the 4 phases of interest: baseline, early learning, late learning and initial washout trials. The average across participants is shown, and error bars indicate standard errors of the mean. A horizontal dotted line in (**A**) indicates the angle of − 12°, which is the angle that perfectly compensates for the perturbation. (**C**) Boxplots of the learning speeds of the kicking angle and the APAs during the perturbation period. Each dot represents the learning speed of each participant. A significant difference is indicated as * (*p* < 0.001).

When initially exposed to the rotational perturbation, participants were feedbacked a large angular error of the cursor trajectory in the clockwise direction. After repeated exposure to the perturbation, the kicking angle gradually changed counterclockwise from the baseline angle by the end of the learning phase (baseline, 0.30 ± 0.93 deg; late learning, − 10.85 ± 0.97 deg; *p* < 0.001, *d* = 9.94; Figs. 4, 5A) to allow the cursor to reach the target. In the washout period, where the perturbation was turned off and the actual cursor position was fed back, the kicking angle was gradually modified to a level equivalent to that in the baseline period. The size of the initial kicking angle in the washout period was significantly different from that in the baseline trials (baseline, 0.30 $\pm$ 0.93 deg; initial washout, − 4.2 ± 1.71 deg; *p* < 0.001, *d* = 2.23; Fig. 5A). This aftereffect indicates that participants adapted their goal-directed movements to the novel visuomotor environment. Meanwhile, while the kicking-foot movements were adapted to the perturbation, the APAs were also modified ﻿gradually from the baseline in the same direction of the change in the kicking-foot movements (baseline, 0.66 ± 0.38 cm; late learning, 0.44 ± 0.40 cm; *p* = 0.002, *d* = 0.84; Figs. 4, 5B). However, the size of the APAs in the initial washout period could not be distinguished from that of the baseline APAs (baseline, 0.66 ± 0.38 cm; initial washout, 0.66 ± 0.53 cm; *p* = 0.98, *d* = 0.01; Fig. 5B).

Although the kicking-foot movements and APAs changed in the same direction during the perturbation period, they changed at different speeds. At the beginning of the perturbation period, the kicking-foot movements changed quickly, and the mean kicking angle of the first 10 perturbation trials was significantly different from that at baseline (baseline, 0.30 ± 0.93 deg; initial learning, − 5.03 ± 2.14 deg; *p* < 0.001, *d* = 2.56; Fig. 5A), while the mean of the APAs in the first 10 perturbation trials was not significantly different from that at baseline (baseline, 0.66 ± 0.38 cm; initial learning, 0.75 ± 0.41 cm; *p* = 0.14, *d* = 0.35; Fig. 5B). Additionally, an exponential function was fitted to the kicking angle and the APAs during the perturbation period to evaluate the speed of the change during adaptation^18^. As a result, the APAs were modified more slowly than the kicking-leg movements were adapted (Wilcoxon signed-rank test, *p* < 0.001, *r* = 0.88; Fig. 5C). On the other hand, at the beginning of the washout period, the kicking angle showed an after-effect of learning, while the amplitude of the APAs was not different from that at baseline. The APAs tended to return to the baseline level more quickly than the kicking angle in the washout period.

## Discussion

In this study, we aimed to investigate postural control during the adaptation of goal-directed control of the lower limb. First, APAs were observed in ball-kicking movements and measured as mediolateral displacement of the support-leg COP in this study. The results of the time course of learning demonstrated that APAs gradually changed in parallel with the adaptation of goal-directed kicking movements in a novel visuomotor environment (Fig. 4). Furthermore, APAs were modified more slowly than the adaptation of kicking-leg movements (Fig. 5C).

Overall, the adaptation pattern observed in the kicking movements was consistent with the findings of previous studies on visuomotor adaptation in arm-reaching^7,8^ and kicking movements^9^ showing gradual reduction in the endpoint errors and a remarkable aftereffect in the initial washout trials (Fig. 4). The primary finding in this study was that anticipatory postural control in the support leg changed more slowly than the kicking-leg movements during the adaptation (Figs. 4 and 5C). Our findings are in line with a study by Ahmed and Wolpert, in which APAs were learned more slowly than the dynamics of reaching in arm control^18^. Despite the higher instability in ball-kicking movements than in arm-reaching movements, APAs were modified at a slower rate than kicking-leg movements. Regardless of the effectors used for goal-directed control, the postural system would require more trials to adjust anticipatory postural control compared to that required for adapting goal-directed control. In contrast to the previous study, which demonstrated an aftereffect in the learning of APAs^18^, this study indicated that the amplitude of the mediolateral APAs in the initial washout trials is not different from that at baseline. The slower learning rate and the absence of the aftereffect are hallmarks of learning a novel controller^22,23^. Therefore, our results suggest that the postural system learned a novel controller for anticipatory postural control that is suitable for the adapted kicking-leg movements during the perturbation period.

There seemed to be large individual differences in the learning speed of the kicking angle (Fig. 5C). One possible explanation about this result is that the strategy of learning was different for each participant. Indeed, in an interview after the experiment, some participants reported that they changed their aiming of the kicking direction during the perturbation phase, but others did not realize the perturbation even though the kicking angle significantly changed. This implies that how much participants relied on the explicit and implicit process to learn the kicking-leg movements depended on the individuals. On the other hand, there were no participants who reported that they explicitly changed the strategy of the postural control. Previous studies involving visuomotor adaptation tasks during arm-reaching movements have reported that the explicit strategic learning process progresses more rapidly than the implicit learning process^24,25^. Therefore, we infer that the individual difference of the learning rates of the kicking angle was induced because the learning strategy of kicking-leg movements varied among participants.

When performing ball-kicking movements, the COP of the support leg started to shift from the initial position forwards and ﻿in the direction of the kicking leg approximately 300 ms before the onset of the kicking movement (Fig. 2). The timing of the COP displacement onset is consistent with previous studies demonstrating that the postural muscle of the support leg responded approximately 200–300 ms before the initiation of the kicking movement^3,4^. It was also shown that the COP position before the onset of the kicking-foot movement shifted in the direction of the kicking leg^21^. In contrast, when we initiate forwards walking, the COP moves in a backwards direction and then towards the swing limb. The backwards displacement of the COP contributes to the acceleration of the COM in the forwards direction^1^. Although ball-kicking movements and walking are the same in that both involve swinging the lower limb, the direction of the COP displacement was opposite in the anteroposterior direction. This distinction probably arose from the difference in the motor goal associated with ball-kicking movements and walking. In the current experimental task, participants were instructed to maintain their support foot on the force plate and put their kicking foot next to the support foot after the kicking movement. Conversely, walking requires continuous propulsion of the COM forwards. Hence, the forwards COP displacement in advance of the onset of the kicking movement probably aimed to prevent undesired forwards acceleration of the COM. It is supposed that APAs are modulated depending on the specific goal of the movement.

In addition to the modification of the APAs during the adaptation of the kicking-foot movements, we also observed a leftward change in the COP position at the moment when the kicking-foot movement reached the target distance, approximately 200 ms after the initiation of the kicking-foot movement (Figs. 2 and 3). The direction of the change corresponded to the direction in which the kicking-foot movements and the mediolateral APAs were modified. Such behaviours of the COP would reflect the change in the COM position resulting from the correction of the kicking-foot movements in the mediolateral direction during the adaptation process. These findings also suggest that anticipatory postural control is modulated in response to the demands of maintaining the desired COM position. In the anteroposterior direction, however, the COP position did not show systematic change during the adaptation of the kicking-foot movements, indicating that the primary adjustment occurred in the mediolateral direction.

Furthermore, the COP trajectories during the kicking movements showed larger variability among participants. Several factors may contribute to this observed variability. First, human posture exhibits spontaneous sway even during a quiet stance^26,27^. This inherent postural sway can cause larger variability in the COP movements for each trial. Second, there are also individual differences in postural behaviour. Practicing any kind of sport have been reported to improve postural stability^28^. Although participants in the current study did not have experience with soccer or futsal training, some of them had trained in other sports. It is also known that COP displacement has a negative correlation with the maximal isometric torque of ankle muscles^29,30^. Moreover, the greater requirement of postural stability in a single leg stance leads to strategies of postural control that involve ﻿increasing stiffness, resulting in a smaller COP displacement^31,32^. These factors could contribute to the larger individual differences observed in the APAs during the kicking movements. Indeed, the trend of aftereffects in the APAs was observed in several participants, although the mean behaviour of the APAs did not show aftereffects (Fig. 4). Hence, conducting experiments that can induce a larger learning response, such as introducing a larger size of visuomotor perturbation or dynamic perturbation, would expand our knowledge of the interaction between APAs and goal-directed movements with the lower limb.

## Conclusion

The current study demonstrated that anticipatory postural control is modified in the process of the adaptation of focal goal-directed movements with the lower limb. Moreover, such predictive postural control was changed more slowly than the adaptation of goal-directed movements. These findings suggest that both goal-directed movements and postural control are modified, but at different rates, when adapting to the novel visuomotor environment. Furthermore, the alteration of anticipatory postural control depends on the requirements of voluntary movements. This study contributes to our understanding of the role of the support leg in the process of adapting goal-directed movements with the lower limb and provides insights into the dynamic relationship between postural control and adaptation.

## Methods

### Participants

Nineteen healthy young adults (age = 21.1 ± 2.2 years, height = 166.7 ± 8.3 cm, weight = 59.8 ± 10.3 kg, mean ± standard deviation [SD] including 8 females) participated in this experiment. All participants were right-footed and did not have experience with soccer or futsal training in club activities at the college, high school, or junior high school level. They gave informed consent, and the experimental procedures were conducted in accordance with the Declaration of Helsinki and approved by the Ethics Committee for Human Experimentation at the Graduate School of Human and Environmental Studies, Kyoto University (22-H-45).

### Experimental setup

The experimental setup is illustrated in Fig. 1. Participants stood barefoot with their left leg on a force plate (EFP-S-1.5kNSA13B, Kyowa, Tokyo, Japan) and their hands on their hips with a cloth blocking their sight of their foot. A 27-inch LCD monitor (60 Hz) was placed 80 cm in front of the participants at eye level. During the task, the right foot position on the horizontal plane and the COP position of their left foot were captured in real time and displayed on the monitor for visual feedback as white cursors.

The right foot position was captured by the real-time motion tracking system^9,33^. Trajectories of a rigid body created by four infrared reflective markers attached on the instep of the right foot were recorded at 100 Hz using the three-dimensional optical motion capture system (OptiTrack V100, Natural Point Inc., Oregon, United States). The rigid body coordinates were streamed from Motive 2.0.2 software (Natural Point Inc., Oregon, United States) via NatNet SDK to LabVIEW (National Instruments Austin, TX) in real time. The left foot COP position was also calculated in real time based on the vertical components of the force plate data and displayed by a program developed using LabVIEW. The signal recorded from the force plate was stored at a sampling frequency of 500 Hz on the hard disk of a personal computer by a 16-bit A/D converter (NI-DAQ USB-6229, National Instruments, Austin, TX).

The monitor displayed a target (0.6 cm radius circle), a cursor (0.4 cm radius white circle) representing the right foot position on the horizontal plane and the home position of the right foot (0.75 cm radius green circle; Fig. 1B). In addition, the monitor displayed the COP position of the left foot (0.3 cm radius white circle) and its home position (0.6 cm radius green circle) next to the green circle showing the right-foot home position. The target was displayed above the screen, and the home position of the right foot was 14.2 cm below the target. The distance between the right-foot home position and the target was 60 cm in reality.

### Experimental procedure

Participants performed a ball-kicking movement with their right foot to shoot through a target displayed on the monitor. At the start of each trial, participants moved the cursor representing their right foot position to the home position (within 1.5 cm in reality). The cursor representing the COP location of the left foot and its home position appeared after the participant’s right foot was placed in the home position. Participants were required to hold ﻿their COP cursor within the home position (0.75 cm radius in reality). ﻿This ensured that they returned to a certain position at the beginning of every trial. The target appeared after both cursors of the right foot and the COP were held in the home positions to ensure the participants started each trial with the same posture. After 1.5 s, the COP cursor and its home position disappeared, and a green target circle appeared. After 0.5–1.5 s, the target’s colour turned from green to magenta, and the right-foot home position disappeared, which was the cue that participants should initiate the movement towards the target. ﻿If participants moved before the appearance of the magenta target, the trial was aborted and repeated. Movement speed was incentivized by a “slow” or “fast” message appearing at the end of the trial if the maximum velocity of the right foot was slower than 4.5 m/s or faster than 6 m/s. Participants were instructed to reach the right foot cursor to the target as accurately as possible. They were also asked to keep their support foot on the force plate, shoot the target without backwards movements and put their kicking foot next to the support foot after shooting the target.

All participants were first allowed to become familiarized for 40 trials. ﻿After the familiarization, participants performed 480 trials (30 trials × 16 sets) for the main experiment. These trials were divided into three phases: baseline, perturbation, and washout periods (140, 210, and 130 trials, respectively). Participants rested for 60 s after each set. In the baseline and washout periods, the position of the right foot cursor corresponded to the actual position of the participant’s right foot on the horizontal plane. In the perturbation period, a visuomotor perturbation was introduced without informing participants to examine the process of visuomotor learning^9,25,34^. In the current study, the trajectories of the right foot were rotated by 12° clockwise relative to the actual trajectories (Fig. 1C). The visuomotor rotation angle was equivalent to the angle of modification for the kicking leg in our previous study, in which the trajectories of virtually kicked ball aimed at a target was rotated^9^. This rotation requires a 10.9 cm leftward displacement of the kicking position from the target position to compensate for the perturbation. All the experimental tasks were implemented using a custom-made LabVIEW script.

### Data analysis

#### Analysis of the goal-directed control of the kicking leg

The kicking angle on a given trial was calculated as the angular difference between the vectors of the target and the right-foot position originating from the home position at the moment when the right foot exceeded the target distance as a performance index to evaluate adaptation to the visuomotor rotation task. The mean kicking angle of the first 10 trials of the washout period was quantified as an aftereffect.

The trajectories of the right foot sampled at 100 Hz were used for the analysis of the right foot trajectories on the horizontal plane. The time series of the cursor position was filtered using a zero-lag, fourth-order Butterworth filter with a low-pass filter cut-off of 8 Hz^9^. Movement onset was defined as the moment when the right foot movement exceeded 2.5% of peak velocity. All data were aligned to the movement onset of the right foot. Individual trials were excluded as outliers if the maximum movement distance did not reach the target. This resulted in the omission of < 0.03% of trials (2 trials out of 7680).

#### Analysis of the postural control of the support leg

Postural control was measured from the COP movement, which reflects the net external moment generated about the ankle. The COP position data were calculated from the force plate data, which were sampled at 500 Hz and were low-pass filtered at 10 Hz^35^. Most changes in the kicking-foot movements to compensate for the perturbation appear in the mediolateral direction; thus, we measured the COP movement in the mediolateral direction initiated prior to the right foot movement as anticipatory postural control.

A typical pattern of anticipatory responses in postural muscle activity prior to self-initiated or predictable perturbations started approximately 100 ms before movement onset^36–38^, and anticipatory COP displacements were observed between 50 ms before movement onset and 100 ms after movement onset^18,35^. In the current study, the ﻿APAs were measured as a mediolateral COP displacement at 50 ms before the kicking foot started to move so that the value of the APAs in the support leg could be isolated from contamination from COP displacement caused by kicking-leg movements. The mean APAs of the first 10 trials of the washout period was quantified as an aftereffect.

### Learning speed of the kicking angle and the APAs

To evaluate the speed of the change during adaptation, an exponential function was fitted to the kicking angle and the APAs during the perturbation period as follows:

$$f\left(t\right)=\left(a-b\right)*{e}^{-\beta *t}+b$$

where *t* denotes the trial number during the perturbation period and *b* denotes the mean of the last 50 trials in the perturbation period^18^. The fittings were performed in the least-squares method. We quantified the speed of change as $\beta$ for each participant’s data.

### Statistics

All statistical tests were executed using JASP (https://jasp-stats.org/)^39^. One-way repeated-measures ANOVAs were used to test the difference in the mean kicking angle and the mean APAs between each phase: the last 50 baseline trials (baseline), the initial 10 trials in the perturbation period (initial learning), the last 50 trials in the perturbation period (late learning) and the initial 10 trials in the washout period (initial washout). Once a significant F value was achieved, Bonferroni post hoc tests were performed. We used the Wilcoxon signed-rank test for the comparison of the learning rates of the kicking angle and the APAs during adaptation. In all statistical tests, the difference was assumed to be significant at an α threshold of 0.05, and normality assumptions for the tests were verified with Shapiro–Wilk tests.

## Data Availability

The datasets analysed during the current study are available from the corresponding author upon reasonable request.
